# Cost-effectiveness of Tele-delivered behavioral activation by Lay counselors for homebound older adults with depression

**DOI:** 10.1186/s12888-022-04272-9

**Published:** 2022-10-17

**Authors:** Guoqing John Chen, Mark E. Kunik, C. Nathan Marti, Namkee G. Choi

**Affiliations:** 1 University of Kanas Medical Center, Kansas City, KS USA; 2 HSR&D Center for Innovations in Quality, Effectiveness and Safety, Baylor College of Medicine, Michael E. DeBakey VA Medical Center, Houston, Houston, VA, TX USA; 3 Education and Clinical Center, VA South Central Mental Illness Research, Houston, TX USA; 4 Steve Hicks School of Social Work, University of Texas at Austin, Austin, TX USA

**Keywords:** Depression, Lay counselors, Behavioral activation, Problem-solving therapy, Cost-effectiveness, Incremental cost-effectiveness ratio

## Abstract

**Background:**

Low-income homebound older adults have limited access to psychosocial treatments because of their homebound state and geriatric mental health workforce shortages. Little is known about cost effectiveness of lay-counselor-delivered, videoconferenced, short-term behavioral activation on this study population. The objective of this study was to assess the cost-effectiveness of lay-counselor-delivered, videoconferenced, short-term behavioral activation (Tele-BA) compared to clinician-delivered, videoconferenced problem-solving therapy (Tele-PST) and telephone support calls (attention control; AC) for low-income homebound older adults.

**Methods:**

We performed a cost-effectiveness analysis based on data from a recently completed, 3-group (Tele-BA, Tele-PST, and AC) randomized controlled trial with 277 participants aged 50+. We measured total costs of (1) intervention and (2) outpatient care, ED visits, and inpatient care using the Cornell Services Index. The effectiveness outcome was quality-adjusted life-years (QALY). We used EuroQol’s EQ-5D-5L to assess each participant’s health-related quality of life (HRQoL) at baseline and at 12, 24, and 36 weeks. The end-point measure of cost-effectiveness was the incremental cost-effectiveness ratio (ICER) of (1) Tele-BA versus AC, (2) Tele-PST versus AC, and (3) Tele-BA versus Tele-PST.

**Results:**

Relative to AC, both Tele-BA and Tele-PST are cost-saving treatment options. The ICERs for both Tele-BA and Tele-PST were well below $50,000, the lower-bound threshold for cost-effectiveness. Relative to AC, both Tele-PST, Tele-BA are cost-saving treatment options (i.e. lower costs and more QALYs).

**Conclusion:**

Costs of tele- and lay-counselor-delivered depression treatment are modest and cost effective relative to providing telephone support. Though our results show that Tele-BA may not be cost effective relative to Tele-PST, a clinician-delivered psychotherapy, when a low bound ICER threshold of $50,000 would be used, lay counselors can fill the professional geriatric mental health workforce shortage gap and Tele-BA by lay counselors can improve homebound older adults’ access to evidence-and skills-based, cost effective depression care.

**Trial registration:**

ClinicalTrials.gov identifier: NCT02600754 (11/09/2015).

**Supplementary Information:**

The online version contains supplementary material available at 10.1186/s12888-022-04272-9.

## Background

With unprecedented growth in the number of older adults in the U.S., [1] the number of homebound older adults is increasing rapidly [2]. One study estimated that over a 7-year period, 8.3% of Medicare beneficiaries were persistently homebound (i.e., never or rarely left home during the last month), and 26.2% had a rapid increase in their risk of being homebound [3]. With high COVID-19 fatalities in long-term care facilities, [4] older adults and their family members may also be delaying decisions about institutional care, further increasing the number of older adults who are disabled and homebound.

Although depression tends to be less prevalent among older than younger adults, homebound older adults are an exception. With multiple chronic medical conditions, disability, and hospitalizations restricting their social engagement and activities, older adults who are homebound experience depression at two-to-three times higher rates than their peers who are not homebound [5–8]. Women, racial/ethnic minorities, and more socioeconomically disadvantaged are overrepresented among homebound older adults. [2, 6] Financial stressors are a significant depression risk factor among low-income homebound older adults [9, 10]. In a sample of 2,200 low-income homebound individuals aged 50 + receiving home-delivered meals, 37.8% reported a depression diagnosis [11].

Compared to older adults without depression, those with depression incur higher healthcare costs, with higher symptom severity associated with higher costs [12–14]. Among low-income homebound older adults, depressive symptom severity is also significantly positively associated with emergency department (ED) visits [15]. The most common treatment for geriatric depression is antidepressant medications prescribed by primary care physicians [16]. However, pharmacotherapy’s effectiveness for low-SES older adults with multiple life stressors is limited, [17] while psychosocial treatments (e.g., cognitive-behavioral therapy [CBT], problem-solving therapy [PST]) have been found effective as they offset skill deficits associated with late-life depression, especially in the context of disability [18]. Meta-analyses of psychosocial treatments, especially PST, found that they improve the psychological and functional outcomes for disabled older adults with high comorbidity [19, 20], which, in turn, can reduce excess healthcare costs.

Low-income homebound older adults with depression have responded well to brief, home-based and videoconferenced PST (Tele-PST) delivered by licensed clinicians [21]. Equivalence between videoconferenced and in-person sessions in treatment effects of mental healthcare has been well-established [22]. However, due to professional geriatric mental health workforce shortages, [23] depression treatment that can be delivered by trained lay counselors might be likely to be more scalable and sustainable for these older adults. In our recent randomized clinical trial (RCT), the clinical effectiveness of reducing low-income homebound older adults’ depressive symptoms using brief, videoconferenced behavioral activation (Tele-BA) delivered by bachelor’s-level lay counselors (who did not have mental health training prior to the study) was compared to (1) Tele-PST delivered by licensed clinicians and (2) an attention control (AC) condition (telephone support calls by research assistants). We found that compared to AC, both Tele-BA and Tele-PST were more effective in reducing depressive symptoms, but Tele-PST’s effect size (d = 1.00 [95% CI = 0.73–1.26]) was larger (p < 0.001) than Tele-BA’s (d = 0.62 [95% CI = 0.35–0.89]).^24^ However, Tele-BA’s effects did not significantly differ from Tele-PST’s effects on disability, frequency of social engagement and activities, and satisfaction with participation in social roles [24].

There are no studies examining cost effectiveness of using lay counselors along with telemedicine in management of depression in low-income homebound older adults according to our knowledge. In the present study, using the data from our RCT, we present our findings on the cost-effectiveness of Tele-BA compared to Tele-PST and AC. Along with clinical effectiveness, cost-effectiveness is a significant factor for implementing a psychosocial treatment at a large scale. As the first study to evaluate tele-delivery of depression treatment by lay counselors in the homebound older-adult population, the findings provide insight into the potential for real-world implementation of lay-counselor-delivered geriatric depression treatment.

## Methods

### Participants and setting

Between February 15, 2015 and April 15, 2019, case managers at a large aging service and home-delivered meals agency in Central Texas referred 441 homebound older adults aged 50 + to the study. Of them, we enrolled 277 who met the following inclusion criteria: (1) moderately severe to severe depressive symptoms (24-item Hamilton Depression Rating Scale [HAMD] [25, 26] score ≥ 15); (2) non-Hispanic White, Black/African American, or Hispanic; and (3) English or Spanish proficiency. Exclusion criteria were: (1) high suicide risk, probable dementia, bipolar disorder, psychotic disorder, substance misuse, antidepressant medication intake/modification < 8 weeks, and (2) current participation in any psychotherapy. Each eligible participant was randomly assigned into one of three arms. Detailed descriptions of the study design and methods, participants, and settings are provided elsewhere [24]. The study was registered at ClinicalTrials.gov (NCT02600754; 11/09/2015). The authors’ institutional review boards approved the study. No adverse study-related events were reported.

### RCT groups and interventions

Of the 277 study participants, 90 were assigned to five, one-hour weekly Tele-BA sessions; 93 were assigned to five, one-hour weekly Tele-PST sessions; and 94 were assigned to five, up-to-45min AC telephone support calls. Both Tele-BA and Tele-PST were delivered via a HIPAA-compliant videoconferencing platform. Except for a few participants with their own internet connection and computer, all Tele-BA and Tele-PST participants were loaned a laptop and a wireless card for the sessions and received instruction on how to use them. Tele-BA lay counselors and Tele-PST therapists were embedded in the large aging service agency for care coordination with the agency’s case managers.

#### Tele-BA:

Two bachelor’s-level lay counselors (one with a social work degree, the other with a communications degree) were trained to follow a Tele-BA manual for homebound older adults that the investigators adapted from Lejuez et al.’s BA manual [27]. Tele-BA included psychoeducation about depression and the rationale/theory behind BA and teaching participants in steps of increasing and reinforcing value-based life activities and of decreasing depressive behaviors on a daily basis.

#### Tele-PST:

Two licensed master’s-level social workers followed the PST-PC (primary care) manual [28], including psychoeducation and teaching participants problem-solving coping skills (appraisal and evaluation of specific problems, selecting and implementing the best possible solutions) using the same videoconferencing platform. PST also addresses anhedonia and psychomotor retardation through behavioral activation and increased exposure to pleasant events. Tele-BA and Tele-PST interventionists followed the same protocols for training, clinical supervision, and fidelity monitoring.

#### AC:

A lay-counselor equivalent research assistant provided the telephone support calls. The research assistant engaged participants using techniques such as genuine regard, adding perspective, and facilitating self-expression [29], without any direct coaching on specific coping skills development.

### Cost measurement

The CEA was undertaken from a public healthcare payer perspective. We measured total treatment costs as the sum of the monetary value of all resources consumed in two categories: (1) intervention, and (2) healthcare service utilization. Intervention costs included: interventionist compensation (annual salary plus fringe benefit (at average market rate in Texas for entry-level licensed clinical social workers for Tele-PST therapists and for bachelor’s level social workers for Tele-BA lay counselors and AC call providers.) focusing on interventionist time for training/certification, tele-sessions, and care coordination and AC caller time; clinical supervision; electronic devices (laptop computers for interventionists and participants) and transmission (4G LTE wireless cards); work telephones (for communication with clients and case managers); copying of psychoeducation and other session materials and other supplies; equipment setup, maintenance, and troubleshooting; and travel time and mileage reimbursement for equipment delivery/retrieval (Appendix 1). Since both Tele-BA and Tele-PST were tele-delivered, delivery costs were the same for both treatment modalities except interventionist compensation (i.e., salaries were higher for licensed clinicians than lay counselors). These interventionists spent 0.3 year on the delivery of care and the remaining time was research-related activities. All research-related activities and costs were excluded.

Healthcare utilization data were collected using the Cornell Services Index (CSI) including the numbers of outpatient and ED visits and the number of days hospitalized (inpatient care hereafter). The self-reported CSI has demonstrated validity as a measure of health service use when compared to objective data [30]. The CSI records medication names only, which would not enable us to estimate medication cost. Therefore, we calculated the total costs of outpatient care, ED visits, and inpatient care using the following steps: First, we calculated the number of outpatient care and ED visits and the number of days of inpatient care for each participant in each RCT group over the trial period. Second, we calculated treatment cost at the participant level by multiplying the number/days of each service used by the unit cost for outpatient care, ED visit, or inpatient care. The unit costs were based on Medicare’s average reimbursement amounts in the national Medical Expenditure Panel Survey [31] to increase the generalizability of the findings across the U.S.

We calculated total cost for each participant by totaling the costs of intervention, outpatient care, ED visits, and inpatient care. All cost data were adjusted to constant U.S. dollars in 2019 according to the Consumer Price Index.

### Effectiveness measurement

The effectiveness outcome was quality-adjusted life-years (QALY) from baseline to 36 weeks. We used EuroQol’s EQ-5D-5L [32] to assess each participant’s health-related quality of life (HRQoL) at baseline and at 12, 24, and 36 weeks. Participants rated (no, slight, moderate, severe problems or extreme problems/unable to function) their health status in five dimensions: mobility, self-care, usual activity, pain/discomfort, and anxiety/depression. Composite EQ-5D-5L scores were estimated based on participants’ responses using methods described in EuroQol Research Foundation [31] Devlin and Krabbe [33] and Pickard et al. [34] QALYs were estimated based on baseline and follow-up EQ-5D-5L scores using the area under the curve method recommended by Glick et al., [35] in which variations in the length of the follow-up time period for each study participant were taken into account.

### Cost-effectiveness measurement

The end-point measure of this cost-effectiveness study was the incremental cost-effectiveness ratio (ICER) of (1) Tele-BA versus AC, (2) Tele-PST versus AC, and (3) Tele-BA versus Tele-PST over the trial period, which was up to 36 weeks after baseline. The ICERs were estimated as the difference between groups in mean total costs divided by the difference in mean total QALY.

### Data Analysis

We used one-way ANOVA (with Bonferroni corrections for follow-up tests) and Pearson χ square tests to examine participants’ baseline characteristics by RCT group. We used general linear modeling (GLM) with Poisson distribution and log link function, adjusting for over-dispersion by using the scaled Pearson χ square method, to examine the statistical significance of the mean differences in the numbers of outpatient visits, ED visits, and inpatient care days by RCT group. We used GLM with identity link function to examine the statistical significance of the mean differences in costs and QALYs among the three RCT groups. Statistical significance was set at p < 0.05.

We performed a cost-effectiveness analysis by calculating ICER using the intent-to-treat principle as follows: First, we computed the incremental cost (i.e., mean differences in total costs) between RCT groups over the trial period by subtracting the mean total costs for the AC group from the mean total costs for Tele-BA and Tele-PST groups, respectively, and subtracting the mean total costs for the Tele-PST group from the mean total costs for the Tele-BA group. Second, we calculated incremental effectiveness as the net differences in QALYs between the study groups over the trial period in the same manner, i.e., by subtracting the mean QALY utility value for the AC group from the mean QALY utility value for Tele-BA and Tele-PST groups, respectively, and subtracting the mean QALY utility value for the Tele-PST group from the mean QALY utility value for the Tele-BA group. Finally, using the AC group as the base case, we divided incremental costs by incremental effectiveness to derive ICER. [36] The ICER values derived from this study were compared against a conventional cost-effectiveness threshold value of $50,000 per QALY gained, which is considered a lower bound for ICER (i.e., ICER values not exceeding $50,000 were considered cost effective) in US [37]. The sampling uncertainty of ICER estimates were examined by a bootstrap method [38]. Because we employed a 36-week time horizon (i.e., final follow-up assessment at 36 weeks), no discounting was required.

To examine the robustness of ICER estimated with intent-to-treat analysis, we performed the following six sensitivity analyses with: (1) age as a covariate (given the group difference in participant age); (2) participants with at least one (12-, 24-, and/or 36-week) follow-up assessment (N = 260, after excluding 17 participants with baseline data only); (3) participants with baseline and 36-week follow-up data (N = 222, completer analysis); (4) adjusted MEPS unit costs for Medicare beneficiaries aged 50+; (5) adjusted MEPS unit costs for dual Medicare and Medicaid beneficiaries aged 50+; and (6) adjusted MEPS unit costs for all those aged 50+.

## Results

### Participant characteristics

Table 1 shows no baseline differences among the three RCT groups on demographic and clinical characteristics except for age. Tele-PST participants were about 3 years younger than Tele-BA and AC participants.

**Table 1:** Participant Characteristics at Baseline

		Tele-BA	Tele-PST	Attention Control (AC)	***F***_***(df)***_ ***/χ*** ^***2***^_***(df)***_	***P*** Values
		N = 90	N = 93	N = 94		
Age (yrs), M (SD) 1		68.7 (9.5)a	65.5 (8.1)b	68.4 (8.7)c	4.12_(2,274)_	0.017^2^
Gender (%)					0.85_(2)_	0.655
	Female	73.3	67.7	68.1		
	Male	26.7	32.3	31.9		
Race/ethnicity (%)					1.91_(4)_	0.753
	Non-Hispanic White	40.0	44.1	38.3		
	Non-Hispanic Black	32.2	30.1	27.7		
	Hispanic	27.8	25.8	34.0		
Living alone (%)		51.1	45.2	53.2	1.30_(2)_	0.523
Education (%)					1.22_(6)_	0.057
	<High school	23.3	19.4	36.2		
	High school diploma	21.1	11.8	16.0		
	Some college/Associate’s degree	34.4	37.6	27.7		
	Bachelor’s degree or higher	21.1	31.2	20.2		
Household income (%)					11.35_(6)_	0.078
	Up to $15,000	54.4	45.2	62.8		
	$15,001-$25,000	24.4	26.9	27.7		
	$25,001-$35,000	13.3	16.1	5.3		
	$35,001 or higher	7.8	11.8	4.3		
Self-rated financial status (%)					1.84_(4)_	0.766
	Cannot make ends meet/Just manage to get by	82.2	82.8	84.0		
	Have enough to get along, even a little extra	14.4	16.1	14.9		
	Money is not a problem; can buy anything I want	3.3	1.1	1.1		
No. of chronic illnesses (0–9), M (SD)		3.6 (1.6)	3.9 (1.6)	3.8 (1.7)	0.97_(2,274)_	0.381
No. of ADL impairment (0–6), M (SD)		1.8 (1.5)	1.9 (1.6)	1.9 (1.6)	0.10_(2,274)_	0.905
No. of IADL impairment (0–6), M (SD)		2.7 (1.3)	3.2 (1.4)	3.0 (1.6)	2.32_(2,274)_	0.100
Pain rating (0–10), M (SD)		5.4 (2.9)	4.8 (2.7)	4.7 (3.2)	1.63_(2,274)_	0.198
Hamilton Depression Rating Scale score		23.3 (5.7)	22.7 (5.7)	22.9 (5.7)	0.28_(2,274)_	0.753

^1^M (SD) = Mean (standard deviation)

^2^Bonferroni-corrected follow-up comparisons: a > b, p = 0.031; a = c, p = 1.000; and b < c, p = 0.055

### Healthcare Use, costs for intervention and Healthcare Services, and QALY gained

Table 2 shows healthcare services used over the 36-week follow-up period by the study participants and the unit costs associated with each type of service use. Healthcare use was highly variable between individuals, as evidenced by the large standard deviations. The three RCT groups were not significantly different in the mean numbers of outpatient and ED visits, but Tele-PST participants had significantly fewer hospital days than AC participants (z=-2.20, p = 0.028). Table3 shows that intervention costs per participant were $904.06 for the Tele-BA group, $980.75 for the Tele-PST group, and $240.91 for the AC group. Mean total costs per participant were highest for AC ($23,193.13) compared to Tele-BA ($20,086.21) and Tele-PST ($16,437.81), which costed the least. The group differences were largely attributable to inpatient care costs, which were highest for AC ($11,100.75), followed by Tele-BA ($8,520.51) and Tele-PST ($4,254.61) per participant.

**Table 2:** Health Utilization Over 36 Weeks and Unit Cost

	Tele-BA		Tele-PST		AC		Unit Cost 1	
	Mean	SD	Mean	SD	Mean	SD	Mean	SD
No. of outpatient visit	18.24	25.80	19.80	29.35	20.67	29.71	$537.20	$1,665.19
No. of ED visit	1.43	3.94	0.95	2.02	1.24	2.44	$600.51	$805.58
Inpatient days	2.43	6.50	1.22	3.81	3.17	7.44	$3378.85	$5,188.29

^1^Estimated from the 2017 Medical Expenditure Panel Survey

**Table 3:** Mean Cost and Effectiveness Over Trial Period by Group

	Tele-BA		Tele-PST		AC	
	Mean	SD	Mean	SD	Mean	SD
Cost						
Intervention 1	$904.06		$980.75		$240.91	
Outpatient visit	$9,800.89	$13,859.57	$10,634.22	$15,764.82	$11,104.01	$15,961.84
ED visit	$860.74	$2,364.76	$568.23	$1,211.97	$747.45	$1,464.85
Inpatient care	$8,520.51	$22,771.92	$4,254.61	$13,332.11	$11,100.75	$26,045.15
Total	$20,086.21	$26,896.61	$16,437.81	$22,549.10	$23,193.13	$34,504.69
Effectiveness						
QALY 2	0.4146	0.1773	0.3811	0.1495	0.3532	0.1804

^1^ Including unit cost of interventionist compensation per person ($22,479 for Tele-BA, $27,154 for Tele-PST, and $22,479 for AC) and other related cost

^2^ Quality-adjusted life-years: Incremental effectiveness

Table3 also shows that in terms of QALY gained, Tele-BA participants had the highest mean score (0.41), followed by Tele-PST (0.38) and AC (0.35) participants. GLM results show that QALYs gained were significantly higher in the Tele-BA than AC group (t = 2.46, p = 0.015, df = 2); however, differences between the Tele-BA and Tele-PST groups (t = 1.34, p = 0.182, df = 2) and the Tele-PST and AC groups (t = 1.12, p = 0.262, df = 2) were not statistically significant.

### Cost-effectiveness

Table 4 shows the results of incremental cost, incremental effectiveness, ICER, and bootstrapping on ICER estimates. Relative to AC, Tele-BA’s and Tele-PST’s incremental costs per participant were -$3,106.92 and -$6,755.32 (i.e., cost saving), respectively. Relative to Tele-PST, Tele-BA’s incremental cost per participant was $3,648.39 higher. Relative to AC, Tele-BA’s and Tele-PST’s incremental QALY gains were 0.06 and 0.03, respectively. Relative to Tele-PST, Tele-BA’s incremental QALY gain was 0.03. The ICER was -$50,601 for Tele-BA versus AC, indicating that Tele-BA was less costly and more effective than AC or AC was dominated by Tele-BA. The ICER was -$242,126 for Tele-PST versus AC, indicating that Tele-PST was less costly and more effective than AC or that AC was dominated by Tele-PST. The ICER was $108,907 for Tele-BA versus Tele-PST, indicating that Tele-BA was more costly, although it was as effective as Tele-PST. Using the ICER threshold of $50,000 per QALY gained, Tele-BA was not cost effective relative to Tele-PST but became cost effective when the higher bound threshold of $150,000 was adopted. The bootstrap results of ICER estimates were consistent with these findings.

**Table 4:** Estimated Incremental Cost Effectiveness Ratio (ICER) in Study Groups

	Tele-BA vs. AC	Tele-PST vs. AC	Tele-BA vs. Tele-PST
	Mean Difference	Mean Difference	Mean Difference
Incremental cost	-$3,106.92	-$6,755.32	$3,648.39
QALY 1	0.0614	0.0279	0.0335
ICER 2	-$50,601	-$242,126	$108,907
Bootstrap 3			
Mean	-$68,729	-$249,834	$62,105
Median	-$49,385	-$179,548	$85,546
SD	$302,564	$2,803,617	$2,237.338
Interquartile Range	$104,395	$312,645	$178.549

^1^ Quality-adjusted life-years: Incremental effectiveness

^2^ Incremental cost-effectiveness ratio

^3^ Bootstrapping on ICER (N = 1000)

Table 5 shows that compared to findings from intent-to-treat analysis, there was no change in ICER direction in any of the six sensitivity analyses. However, the completer analysis shows that compared to Tele-PST, Tele-BA is cost effective.

**Table 5:** Sensitivity Analysis: Estimated Incremental Cost Effectiveness Ratio in Study Groups

	Tele-BA vs. AC	Tele-PST vs. AC	Tele-BA vs. Tele-PST
	Mean Difference	Mean Difference	Mean Difference
**1) Adjusted for age effect**			
Incremental cost	-$2,897.77	-$8,149.94	$5,252.17
QALY 1	0.0608	0.0329	0.0279
ICER 2	-$47,660.62	-$247,718.54	$188,249.98
**2) Participants with at least one follow-up data (N = 260)**			
Incremental Cost	-$3,825.29	-$7,723.00	$3,897.51
QALY	0.0585	0.0226	0.0359
ICER	-$65,390	-$341,726	$108,571
**3) Participants with complete data (N = 222** ^3^ **)**			
Incremental Cost	-$7,473.19	-$9,842.03	$2,368.84
QALY	0.0746	0.0217	0.0529
ICER	-$100,177	-$453,550	$44,780
**4) Using MEPS** ^4^ **unit cost for Medicare beneficiaries aged 50+**			
Incremental Cost	-$3,078.54	-$9,842.03	$2,368.84
QALY	0.0614	0.0279	0.0335
ICER	-$50,144	-$240,671	$108,534
**5) Using MEPS** **unit cost for Medicare and Medicaid dual beneficiaries aged 50+**			
Incremental Cost	-$2,849.42	-$6,214.74	$3,365.32
QALY	0.0614	0.0279	0.0335
ICER	-$46,407	-$222,751	$100,457
**6) Using MEPS** **unit cost for all those aged 50+**			
Incremental Cost	-$6,624.95	-$11,393.69	$4,768.74
QALY	0.0614	0.0279	0.0335
ICER	-$107,898	-$408,376	$142,350

^1^Quality-adjusted life-years: Incremental effectiveness

^2^Incremental cost-effectiveness ratio

^3^Participants with complete baseline and 36-week follow-up data

^4^Medical Expenditure Panel Survey:

a) unit cost of 50 or over: outpatient $495.23; ER $590.69; and Hospitalization $3120.69/day

b) unit cost of dual status of Medicare and Medicaid: outpatient $518.37; ER $579.47; and

Hospitalization $3378.85/day

## Discussion

We compared the cost-effectiveness of brief Tele-BA delivered by bachelor’s-level lay counselors to brief Tele-PST delivered by licensed clinicians and AC (telephone support calls) by lay-counselor equivalent research assistants. We found that compared to AC, the costs of both Tele-BA and Tele-PST were lower largely due to fewer inpatient care days and QALY outcomes were better in both the Tele-BA and Tele-PST groups. The ICERs for both Tele-BA and Tele-PST were well below $50,000, the lower-bound threshold for cost-effectiveness [37], indicating that Tele-BA and Tele-PST were cost-saving treatment options (i.e., less costly and more effective than AC). Relative to Tele-PST, Tele-BA was not cost-effective when the conventional ICER threshold of $50,000 was used, and at the cost-effectiveness borderline when ICER threshold of $100,000 was used [37]. However, if the higher bound threshold of $150,000^38^ is adopted, Tele-BA was cost effective relative to Tele-PST. In sensitivity analysis with the completer cases, Tele-BA was also cost effective relative to Tele-PST, though potential selectivity bias in completer analyses may have to be considered. More research is needed to understand reasons for the discrepancy between the intent-to-treat and completer analyses. Additional analysis showed no significant difference in baseline characteristics between completers and noncompleters, suggesting that there might be some unobserved factors. The findings show that both Tele-BA and Tele-PST would be cost effective relative to AC; however Tele-BA would not be cost effective relative to Tele-PST when the lower bound threshold of $50,000 was used. The reasons of why study subjects in the Tele-BA group experienced more hospital days but higher QALYs relative to the Tele-PST is unclear. The potential causes of these findings might be severity of illnesses that we did not measure and other unobserved factors in both Tele-BA and Tele-PST groups, which would be subject to further research.

Overall, these findings are congruent with the RCT’s clinical outcomes that both Tele-BA and Tele-PST were superior to AC in reducing depressive symptoms. Tele-PST was superior to Tele-BA in effect sizes [24]. The higher effect size for Tele-PST than Tele-BA was largely driven by its higher response rate (i.e., 50+% reduction in HAMD score), given that remission rates did not differ significantly between the two interventions. Tele-BA was also as effective as Tele-PST in reducing disability and increasing social engagement and activities and satisfaction with participation in social roles [24].

Our findings differ from those of an RCT in the U.K., which found that nonclinician-delivered BA was noninferior to clinician-delivered CBT in reducing depressive symptoms among those aged 18 + with major depression and was also more cost effective [39]. The divergent findings may be due to differences in study samples, which in our study was disabled homebound older adults who had multiple chronic conditions and high rates/costs of healthcare service uses.

In a rapidly aging society, growing numbers of homebound older adults are especially in need of improved access to mental health services. Despite their higher rates of depression, older adults who receive home- and community-based services (HBCS) are no more likely than their peers who do not receive HBCS to receive psychiatric services, suggesting unmet depression needs [40]. Although tele-delivered mental health services have become more available during COVID-19, low-income homebound older adults still face barriers to accessing mental health services due in part to geriatric mental health workforce shortages as well as lack of access to electronic devices and internet connectivity. Though our results show that Tele-BA may not be as cost effective as Tele-PST, a clinician-provided psychotherapy, given current and projected clinician shortages, lay counselors can fill the professional geriatric mental health workforce shortage gap, and brief Tele-BA by lay counselors can improve homebound older adults’ access to evidence-based, cost effective depression care.

Our results should be interpreted with caution in light of study limitations. First, we relied on self-reported information to obtain data on healthcare service use. The self-reports may have led to data inaccuracies, although there were no practical alternatives. The accuracy of the medical utilization estimates in future studies could be enhanced by linking with Medicare and Medicaid claims data since the majority of homebound patients are enrolled in Medicare and/or Medicaid. Second, the time horizon of the cost-effectiveness analysis is limited by the duration of the trial (36 weeks). Since intervention costs occur early, it is possible that additional benefits could accrue beyond 36 weeks. Consequently, we may have under-estimated the potential long-term cost-effectiveness or cost-saving of Tele-BA by lay counselors for homebound older adults with depression. Third, the study is based on data collected in a single randomized controlled trial in a single geographic area, therefore, may not be generalizable to other areas. Fourth, we were not able to estimate medication cost due to lack of medication data on the CSI utilization form. The total cost might be underestimated. Given the limitations of the current study, the findings should be replicated in other areas, employing more sensitive instruments to measure changes in health-related quality of life, optimized cost data collection, and using extended follow-up periods to better capture both costs and health outcomes over time.

Despite these limitations, this study demonstrates that the costs of tele-delivered, skills-based depression treatment provided by bachelor’s-level lay counselors embedded in an aging-service agency are modest, even when all tele-delivery equipment and transmissions costs are included. Relative to AC (or usual care ), the treatment option delivered by lay counselors is a cost-saving or cost-effective strategy than providing telephone support. Given the shortages of geriatric mental health clinicians, this study shows that lay counselors can be trained to deliver evidence-based treatments with good results. While more research is needed, Tele-BA’s clinical effectiveness and cost-effectiveness appear to be sufficient to be considered as a more scalable and more sustainable alternative to clinician-delivered treatments in management of depression in the homebound older-adult population. Medicare and Medicaid reimbursement of lay-provider-delivered treatment sessions is needed to scale Tele-BA for growing numbers of homebound older adults.

## Electronic supplementary material

Below is the link to the electronic supplementary material.

Supplementary Material 1: Estimated Cost of Tele-delivery

## Data Availability

The datasets generated during the current study are not publicly available; however, reasonable data request will be reviewed and granted by the corresponding author.
