# Supplementary material for: Cost-effectiveness of Tele-delivered behavioral activation by Lay counselors for homebound older adults with depression
Source: BMC Psychiatry. 2022 Oct 17;22:648. doi: 10.1186/s12888-022-04272-9 (PMC9574809; doi:10.1186/s12888-022-04272-9)
Supplement: Supplementary file 1 — Supplementary Material 1: Estimated Cost of Tele-delivery [file 12888_2022_4272_MOESM1_ESM.docx]

**Appendix 1. Estimated Cost of Tele-delivery**

|  | Tele-BA | Tele-PST | AC |
| --- | --- | --- | --- |
| Tele-BA/Tele-PST interventionist salary & AC caller compensation | $44,959.20 | $54,309.06 | $22,479.60 |
| Laptops for providers (n=18) | $5,600.00 | $5,600.00 | $0.00 |
| Laptops for participants (n=4) | $2,800.00 | $2,800.00 | $0.00 |
| Wireless card (n=18) | $5,400.00 | $5,580.00 | $0.00 |
| Videoconferencing-platform | $3,800.00 | $3,800.00 | $0.00 |
| Training/supervision of interventionists | $8,300.00 | $8,300.00 | $0.00^1^ |
| Cellphones for interventionists (n=4) | $320.00 | $320.00 | $160.00 |
| Copying and supplies | $4,500.00 | $4,650.00 | $0.00 |
| Other^2^ | $4,410.00 | $4,557.00 | $0.00 |
| **Subtotal (without salary)** | $36,406.03 | $36,900.35 | $165.81 |
| **Total** | $81,365.23 | $91,209.41 | $22,645.41 |
|  |  |  |  |
| N | 90 | 93 | 94 |
| Intervention cost per capita | $904.06 | $980.75 | $240.91 |

^1^AC callers received <2-hour training and the training costs were included in their compensation.

^2^Equipment setup, maintenance, and troubleshooting ($50 hourly x 68.6 hours); and travel time and mileage reimbursement for equipment delivery and retrieval (an average of 25 miles for each round trip x $0.58 per mile per Tele-BA or Tele-PST participant).
